# Supplementary figures and images for: Inhibition of SHP-1 activity by PKC-θ regulates NK cell activation threshold and cytotoxicity
Source: eLife. 2022 Mar 8;11:e73282. doi: 10.7554/eLife.73282 (PMC8903836; doi:10.7554/eLife.73282)

# Figure 1- source data 1

**A**

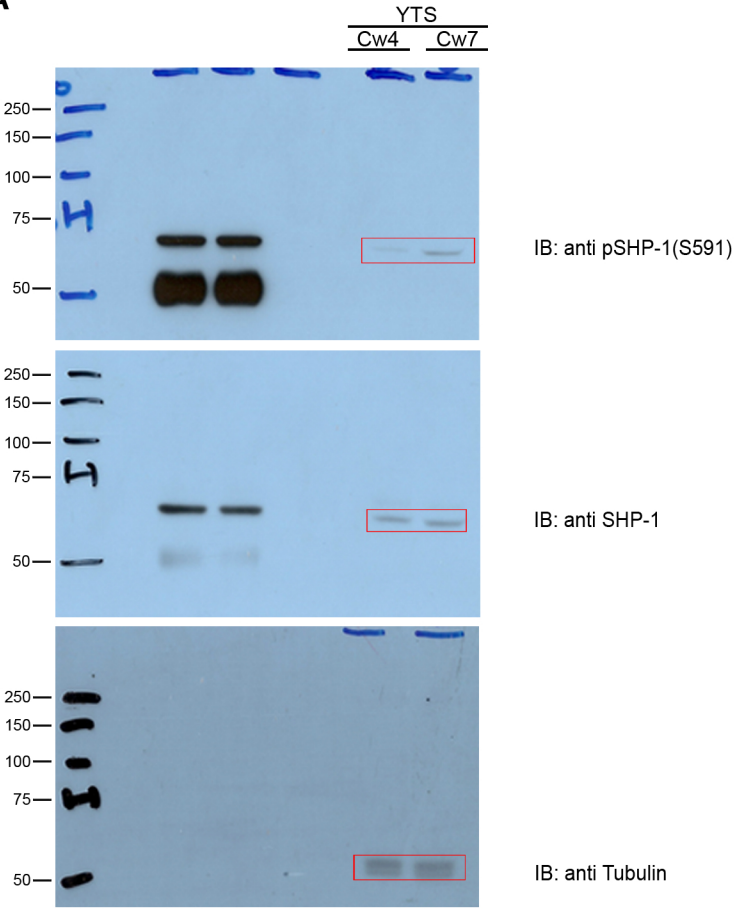

**B**

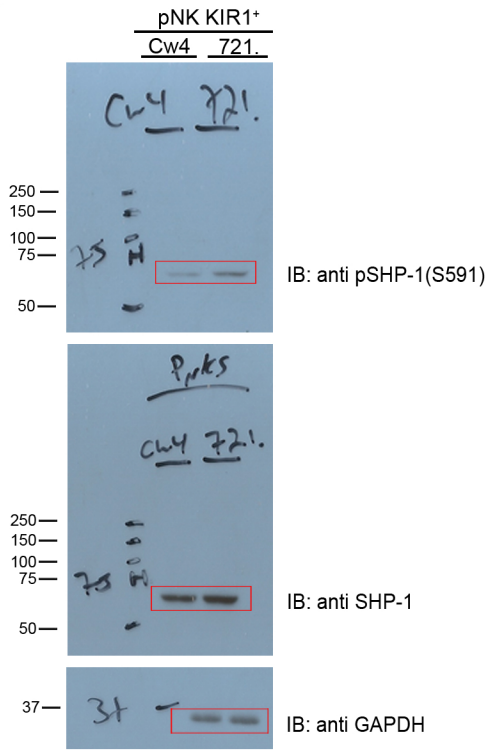

**C**

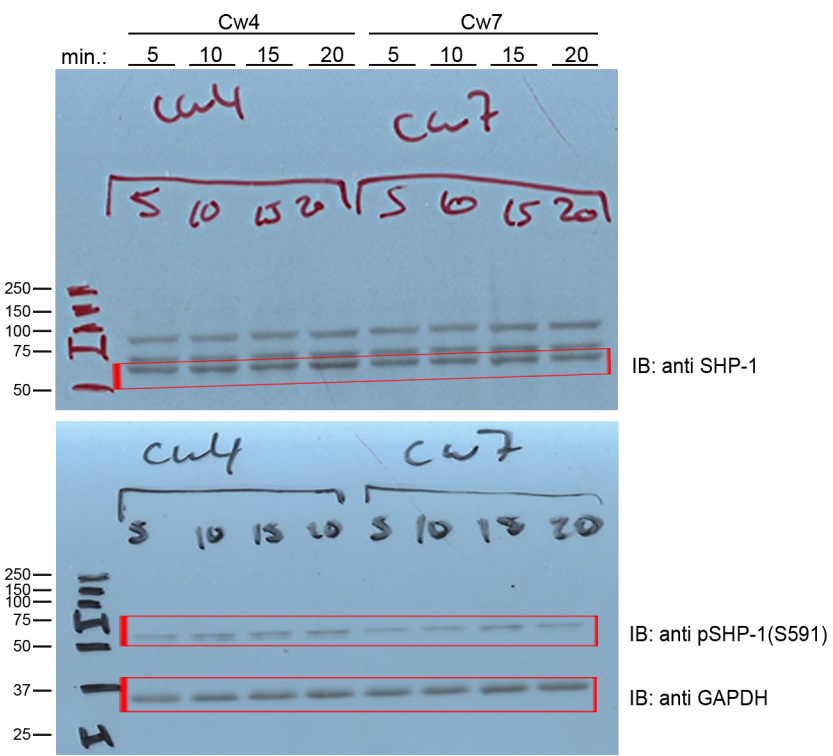

Supplement: Figure 1—source data 1. [file elife-73282-fig1-data1.pdf]

# Figure 4- source data 1

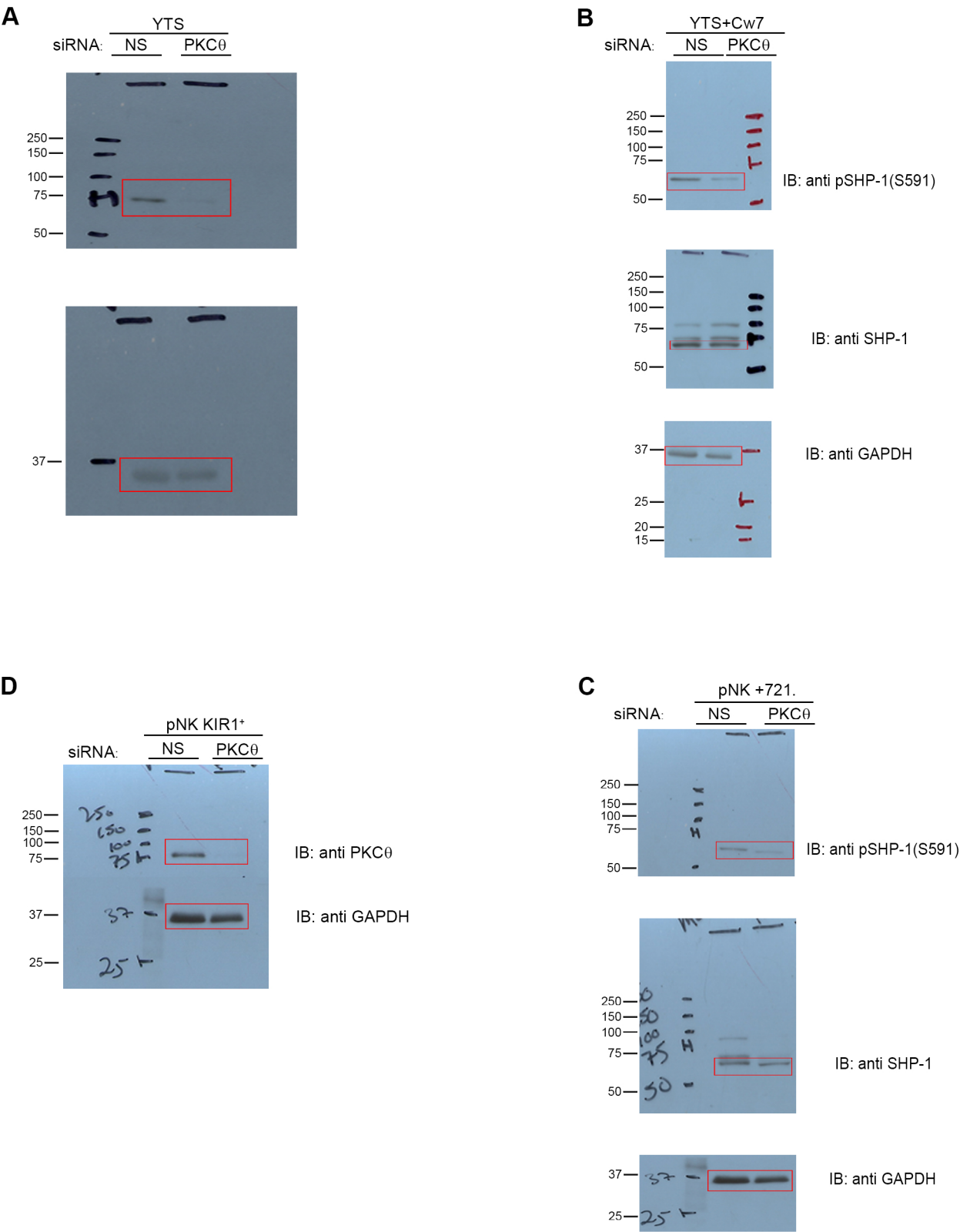

Supplement: Figure 4—source data 1. [file elife-73282-fig4-data1.pdf]

# Figure 5-Figure supplement 2A-source data 1

A

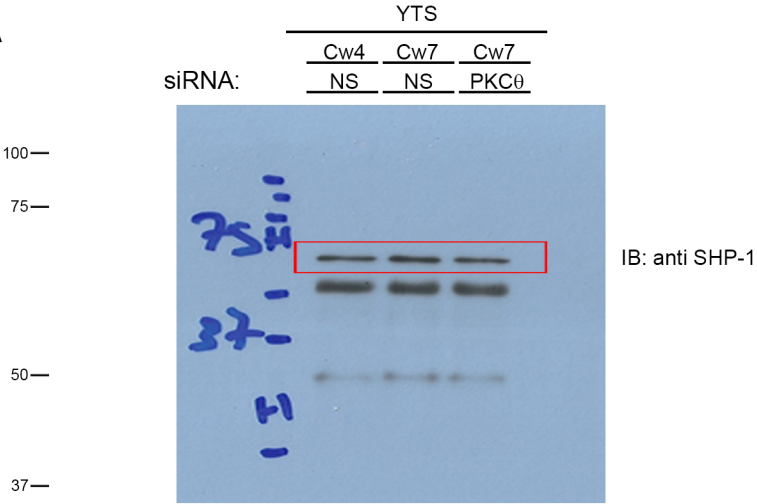

Supplement: Figure 5—figure supplement 1—source data 1. [file elife-73282-fig5-figsupp1-data1.pdf]

# Figure 6- source data 1

A

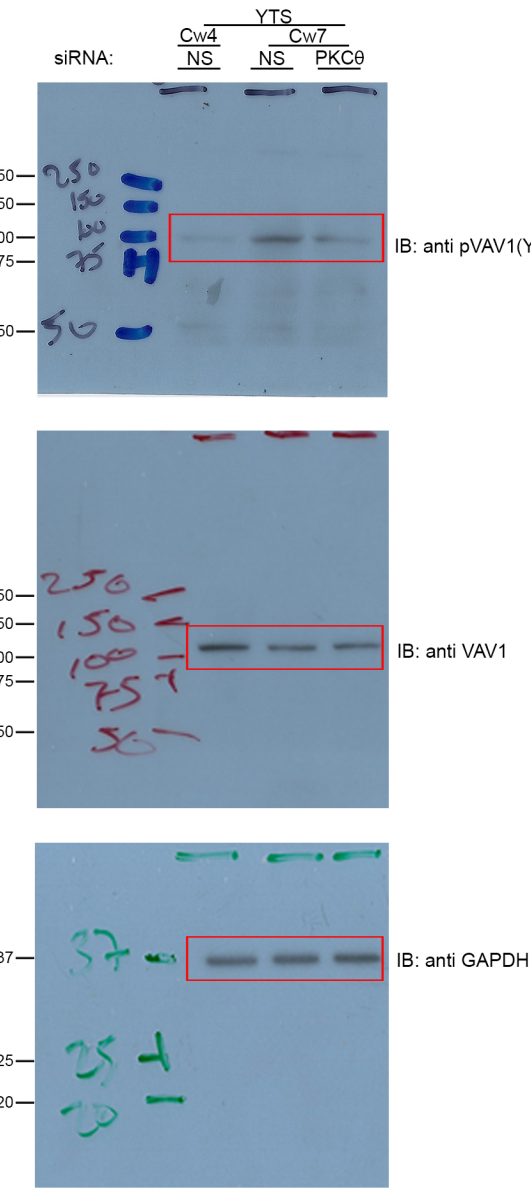

B

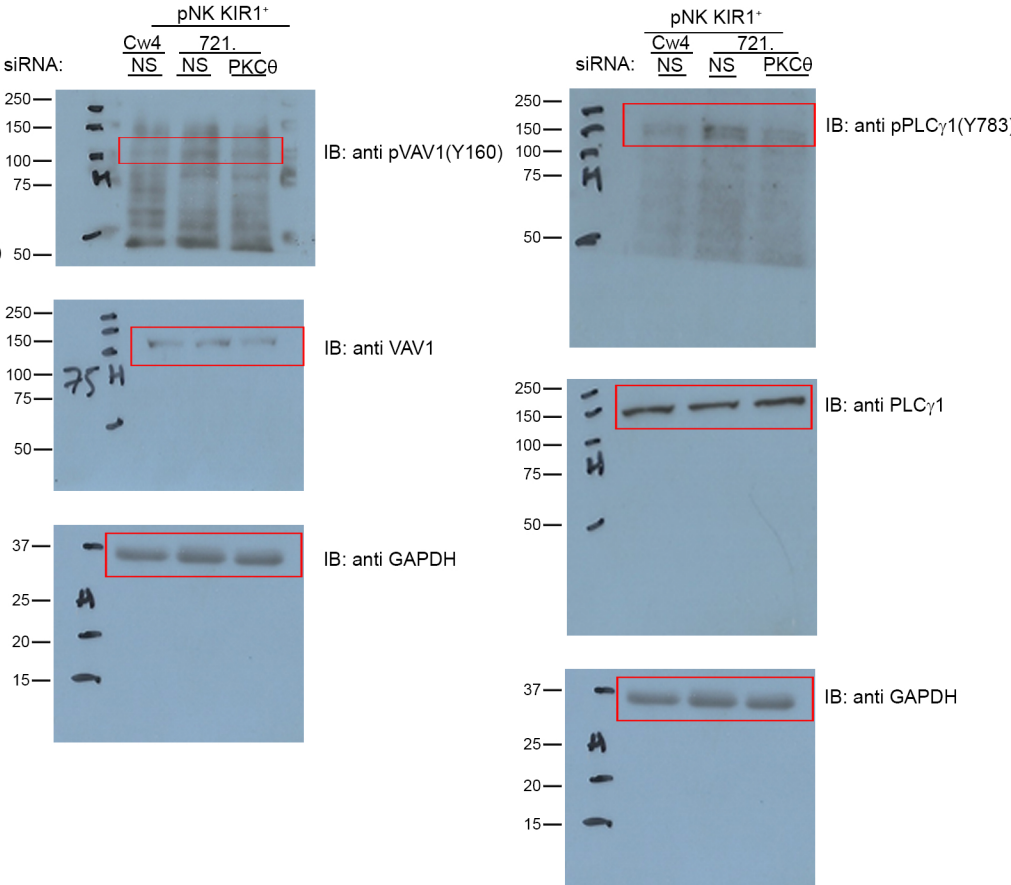

E

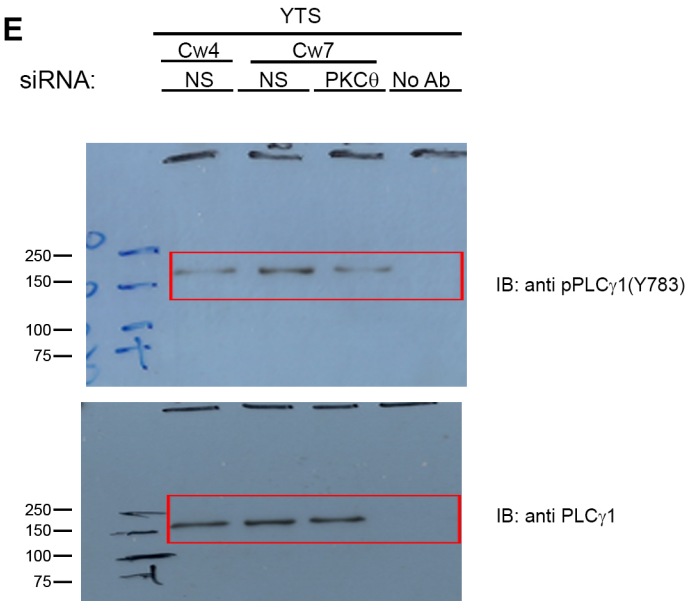

Supplement: Figure 6—source data 1. [file elife-73282-fig6-data1.pdf]

Figure 7- source data 2

D

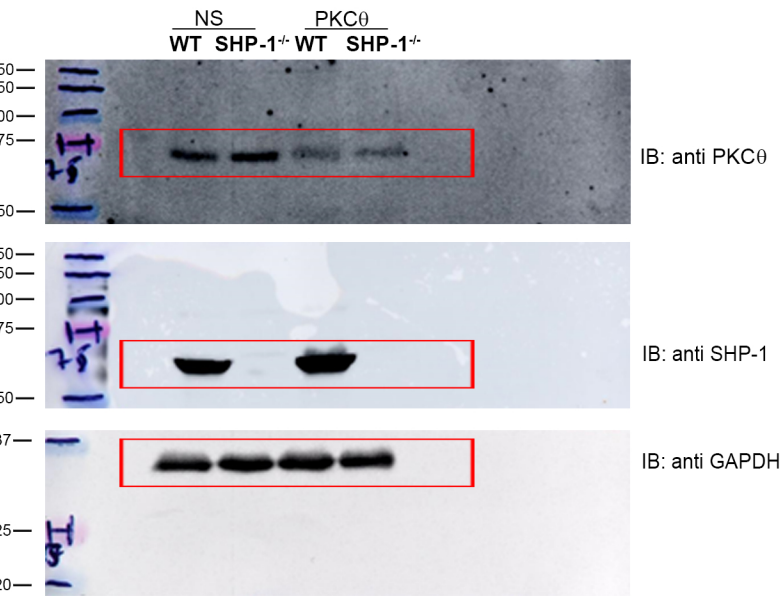

Supplement: Figure 7—source data 2. [file elife-73282-fig7-data2.pdf]
