# Supplementary material for: Inhibition of SHP-1 activity by PKC-θ regulates NK cell activation threshold and cytotoxicity
Source: eLife. 2022 Mar 8;11:e73282. doi: 10.7554/eLife.73282 (PMC8903836; doi:10.7554/eLife.73282)
Supplement: Transparent reporting form [file elife-73282-transrepform1.docx]

***eLife’s* transparent reporting form**

We encourage authors to provide detailed information *within their submission* to facilitate the interpretation and replication of experiments. Authors can upload supporting documentation to indicate the use of appropriate reporting guidelines for health-related research (see [EQUATOR Network](http://www.equator-network.org/%20)), life science research (see the [BioSharing Information Resource](https://biosharing.org/)), or the [ARRIVE guidelines](http://www.plosbiology.org/article/info:doi/10.1371/journal.pbio.1000412) for reporting work involving animal research. Where applicable, authors should refer to any relevant reporting standards documents in this form.

If you have any questions, please consult our Journal Policies and/or contact us: [editorial@elifesciences.org](mailto:editorial@elifesciences.org).

**Sample-size estimation**

- You should state whether an appropriate sample size was computed when the study was being designed
- You should state the statistical method of sample size computation and any required assumptions
- If no explicit power analysis was used, you should describe how you decided what sample (replicate) size (number) to use

Please outline where this information can be found within the submission (e.g., sections or figure legends), or explain why this information doesn’t apply to your submission:

Sample sizes were decided based on practical considerations and experience with the experimental techniques performed. Each experiment included three independent experiments and the values are shown as mean $\pm$ standard errors of the mean were conducted in Microsoft Excel (v14.7.2). The statistical analysis was performed using GraphPad Prism 9.0.1 (GraphPad Software, Inc., USA). P values were calculated using a two-tailed unpaired T test or One sample t-test. Where >2 conditions were compared, a one-way ANOVA or two-way ANOVA with a Tukey post-test was used to calculate P values. Statistical parameters and biological replicates are reported in the figure legends.

**Replicates**

- You should report how often each experiment was performed
- You should include a definition of biological versus technical replication
- The data obtained should be provided and sufficient information should be provided to indicate the number of independent biological and/or technical replicates
- If you encountered any outliers, you should describe how these were handled
- Criteria for exclusion/inclusion of data should be clearly stated
- High-throughput sequence data should be uploaded before submission, with a private link for reviewers provided (these are available from both GEO and ArrayExpress)

Please outline where this information can be found within the submission (e.g., sections or figure legends), or explain why this information doesn’t apply to your submission:

• Number of independent repeats are indicated in all figure legends.

For figure 2,3,5,6C-D and 8 the n value represents the total number of cells/mice used for analysis from three independent experiments.

• For western blot analysis no data were excluded.

• For FACS measurements (Figure 7D), due to fluorescence fluctuations and intrinsic variability the data are represented as relative MFI (mean fluorescence intensity)

**Statistical reporting**

- Statistical analysis methods should be described and justified
- Raw data should be presented in figures whenever informative to do so (typically when N per group is less than 10)
- For each experiment, you should identify the statistical tests used, exact values of N, definitions of center, methods of multiple test correction, and dispersion and precision measures (e.g., mean, median, SD, SEM, confidence intervals; and, for the major substantive results, a measure of effect size (e.g., Pearson's r, Cohen's d)
- Report exact p-values wherever possible alongside the summary statistics and 95% confidence intervals. These should be reported for all key questions and not only when the p-value is less than 0.05.

Please outline where this information can be found within the submission (e.g., sections or figure legends), or explain why this information doesn’t apply to your submission:

P values were calculated using a two-tailed unpaired T test or One sample t-test. Where >2 conditions were compared, a one-way ANOVA or two-way ANOVA with a Tukey post-test (for multiple comparisons) was used to calculate P values. The calculation of inferential statistics (mean $\pm$ standard errors of the mean) is described in the methods section “statistical analysis”.

In Figure 2&5, The raw data points are not represented on the graph. There are large number of data points which would not be informative to display.

(For large datasets, or papers with a very large number of statistical tests, you may upload a single table file with tests, Ns, etc., with reference to sections in the manuscript.)

**Group allocation**

- Indicate how samples were allocated into experimental groups (in the case of clinical studies, please specify allocation to treatment method); if randomization was used, please also state if restricted randomization was applied
- Indicate if masking was used during group allocation, data collection and/or data analysis

Please outline where this information can be found within the submission (e.g., sections or figure legends), or explain why this information doesn’t apply to your submission:

No sample size was predetermined. In in-vitro experiments the groups were defined relative to the control and no randomization was used.

Randomization was used to divide up the animals for in-vivo treatment study.

No blinding was implemented as the researcher who performed the experiments also performed the analysis.

**Additional data files (“source data”)**

- We encourage you to upload relevant additional data files, such as numerical data that are represented as a graph in a figure, or as a summary table
- Where provided, these should be in the most useful format, and they can be uploaded as “Source data” files linked to a main figure or table
- Include model definition files including the full list of parameters used
- Include code used for data analysis (e.g., R, MatLab)
- Avoid stating that data files are “available upon request”

Please indicate the figures or tables for which source data files have been provided:

Figure 1: the representative blots are provided in .PDF format

Figure 2: source data are provided in .xlsx format

Figure 3 + supplements: source data for all graphs in .xlsx format and the representative blots are provided in .PDF format

Figure 4 + supplements: source data for all graphs in .xlsx format and the representative blots are provided in .PDF format

Figure 5 + supplements: source data for all graphs in .xlsx format

Figure 6 + supplements: source data for all graphs in .xlsx format and the representative blots are provided in .PDF format

Figure 7: source data for all graphs in .xlsx format

Figure 8: source data for all graphs showing experimental data in .xlsx format
