# Supplementary material for: Inhibition of SHP-1 activity by PKC-θ regulates NK cell activation threshold and cytotoxicity
Source: eLife. 2022 Mar 8;11:e73282. doi: 10.7554/eLife.73282 (PMC8903836; doi:10.7554/eLife.73282)
Supplement: Peer review history

# Editor report: Editor's evaluation

**DOI:** 10.7554/eLife.73282.sa0

**Authors:** Stipan Jonjic

**Affiliations:** https://ror.org/05r8dqr10 University of Rijeka Croatia

The study by Dr. Barda-Saad and colleagues deals with natural killer (NK) cells activation that depends on a balance between stimulatory and inhibitory signals. Inhibition of NK cells results from the recruitment of the protein tyrosine phosphatase, SHP-1, which dephosphorylates vital signaling molecules and thus reverses NK cell activation. This study describes how PKC regulates the activity of SHP-1 in the early stages of activation and inhibition of NK cell synapses and, by doing so, regulates their adaptation to the microenvironment in which they act.

# Decision letter

**DOI:** 10.7554/eLife.73282.sa1

**Affiliations:** https://ror.org/05r8dqr10 University of Rijeka Croatia; France

In the interests of transparency, eLife publishes the most substantive revision requests and the accompanying author responses.

**Decision letter after peer review:**

Thank you for submitting your article "Inhibition of the SHP-1 activity by PKC-θ regulates NK cell activation threshold and cytotoxicity" for consideration by *eLife*. Your article has been reviewed by 3 peer reviewers, one of whom is a member of our Board of Reviewing Editors, and the evaluation has been overseen by Tadatsugu Taniguchi as the Senior Editor. The following individual involved in review of your submission has agreed to reveal their identity: Emilie Narni-Mancinelli (Reviewer #3).

The reviewers have discussed their reviews with one another, and the Reviewing Editor has drafted this to help you prepare a revised submission.

Essential revisions:

I am pleased to inform you that the reviewers concluded that your paper provides novel insights into the natural killer (NK) cells signaling following activation and inhibition. By demonstrating how PKC downregulates SHP-1 activity at the early stages of activating NK synapse, and at the later stages of the inhibitory NK cell synapse, your study ilustrates mechanism that enables rapid and secure NK cell surveillance in the tumor microenvironment. The reviewers also agreed that your findings will be of interest to scientists in the field of NK cell immunology and that the major claims in the manuscript are well supported by the data. Thus your paper is important and generally suitable for publication in *eLife*.

However, although enthusiastic with the bulk results, the reviewers pointed to one specific aspect that need to be clarified and extended. In the experiment aimed to explore in vivo whether NK cells silenced for PKC−θ have a lower anti-tumor cytotoxicity due to enhanced SHP-1 activity, targets cells that do not exhibit activating signals and thus for which PKC−θ-mediated SHP-I phosphorylation may not be critical, were used. In other words, the reviewers are requesting that you analyse the in vivo growth control of 721-cw7 tumor cell killing by PKC−θ-silenced NK cells.

*Reviewer #1:*

In the study by Ben-Shmuel et al., the mechanism by which SHP-1 is regulated in NK cells is described. By using YTS-2DL1 cell line and primary NK cells from human donors, they show that phosphorylation of the S591 residue of SHP-1 in NK cells by PKC-θ promotes the inhibited SHP-1 state. They show that the phosphorylation of SHP-1 increases along with the progression of the inhibitory NK cell immunological synapse (NKIS) while slightly decreasing during the progression of the activating NKIS. By silencing PKC-θ the authors show that SHP-1 stays in active conformation, and this causes reduced NK cell activation and cytotoxicity. NK cells treated with PKC-θ siRNA show diminished tumor control, confirming that regulation of SHP-1 through PKC-θ impacts NK cell cytotoxicity and their capacity for in vivo tumor surveillance. The study is going to have an important impact in the field. Altogether, the authors provide evidence of a molecular pathway that sustains the NK cell activation threshold through suppression of SHP-1 activity.

*Reviewer #2:*

The manuscript by Ben-Shmuel and colleagues investigates the role of SHP-1 phosphorylation at Serine 591 in NK cell responses. Building on a solid track record of investigating NK cell signaling events, the group of Mira Barda-Saad nicely shows that the S591 phosphorylation of SHP-1 is dynamic and correlates with NK cell activation and inhibition and with the activity of SHP-1 as demonstrated by a FRET probe and a phosphatase assay. They convincingly demonstrate that PKC-θ is the kinase responsible for this phosphorylation. Knock-down of PKC-θ thereby increases SHP-1 activity, promotes the dephosphorylation of SHP-1 targets such as Vav-1 and PLCg1 and more importantly reduces the killing of MHC-1 negative or mismatch targets. These data demonstrate that SHP-1 also plays an important role during NK cell activation and that keeping SHP-1 in an inactive conformation by PKC-θ-mediated phosphorylation is essential for NK cell-mediated anti-tumor responses. The authors use the NK cell line YTS, but also primary human NK cells sorted for KIR2DL1 expression. Overall, their claims are supported by their data. In addition to a few technical remarks, I would suggest focussing more on the role of PKC-θ-mediated SHP-1 phosphorylation during activating NK cell interactions in figures 7 and 8 by using 721-Cw7 targets for the different YTS mutants and transfectants. This would strengthen the novel point that SHP-1 plays an important role during NK cell activation.

1. In figure 7D the authors should also use the 721-Cw7 as targets for the SHP-1 ko and PKC-θ-knock-down YTS cells to demonstrate that the reduction of killing in the absence of PKC-θ is dependent of the presence of SHP-1.

2. The same applies to figure 8, where it would be more informative to see that PKC-θ-knock-down inhibits NK cell-mediated anti-tumor responses against 721-Cw7 cells and that this may be rescued by SHP-1 deletion.

*Reviewer #3:*

Ben-Shmuel and colleagues investigated the mechanisms by which the catalytic activity of SHP-I, a tyrosine phosphatase that is a critical negative NK cell regulator, is regulated during NK cell activation and inhibition. They found that activating NK cell interactions promote a rapid SHP-1 S591 phosphorylation that remains stable overtime, whereas minimal SHP-1 S591 phosphorylation was observed during induced inhibitory interactions. It is only at late time points that inhibitory signals induce high SHP-1 S591 phosphorylation. Such phosphorylation is required to inhibit SHP-1 and NK cell activity, as NK cells expressing a SHP-1 phosphor-mimetic serine to aspartic acid residue substitution (SHP-1 S591D) exhibit increased anti-tumor NK function relative to WT SHP-1-expressing cells (Ben-Shmuel et al., 2020). The authors showed by confocal microscopy an accumulation of phosphorylated SHP-1 S591 at the immunological synapse upon interaction with activating NK cell target cells. SHP-I activity is associated to conformational changes (Wang et al., 2011). The authors further used a SHP-1 Förster resonance energy transfer (FRET) bio- sensor that they have previously developed (Matalon et al., 2018), for which FRET activity is observed when the N and C terminus of the protein are closed, masking the catalytic site. FRET experiments showed that SHP-I is in a conformational inactive state at the immunological synapse as soon as the first 5 minutes of activating NK cell interactions and only after long lasting inhibitory interactions. Ben-Shmuel and coworkers further address by which mechanism SHP-I phosphorylation was achieved. Because the serine kinase PKC−θ was suggested to participate in murine NK cell activity and because PKC−θ localizes to the cytolytic NK synapse during early activation, the authors assessed its possible interplay with SHP-1 in NK cells. They showed that PKC−θ is localized at the immunological synapse early after activating NK cell contact and at late point upon inhibitory contacts. The silencing of PKC−θ limits SHP-1 S591 phosphorylation, SHP-I conformational status and enzymatic activity at the immunological synapse. Finally, they showed that PKC−θ-mediated phosphorylation of SHP-I tunes NK cell activation in vitro. in vivo, PKC−θ silencing of NK cells does not affect their anti-tumor immunity against tumor cells that engage inhibitory NK cell receptors.

The conclusions of this paper are mostly well supported by data, but one aspect need to be clarified and extended. The authors attempted to address in vivo if NK cells silenced for PKC−θ have lower anti-tumor cytotoxicity due to enhanced SHP-1 activity. However, in the final in vivo experiment they analyzed the rejection of targets cells that do not exhibit activating signals and thus for which PKC−θ-mediated SHP-I phosphorylation may not be critical. It is of most interest to evaluate the in vivo growth control of cw7 tumor cell killing by PKC−θ-silenced NK cells.

# Reply: Author response

**DOI:** 10.7554/eLife.73282.sa2

Reviewer #2:

The manuscript by Ben-Shmuel and colleagues investigates the role of SHP-1 phosphorylation at Serine 591 in NK cell responses. Building on a solid track record of investigating NK cell signaling events, the group of Mira Barda-Saad nicely shows that the S591 phosphorylation of SHP-1 is dynamic and correlates with NK cell activation and inhibition and with the activity of SHP-1 as demonstrated by a FRET probe and a phosphatase assay. They convincingly demonstrate that PKC-θ is the kinase responsible for this phosphorylation. Knock-down of PKC-θ thereby increases SHP-1 activity, promotes the dephosphorylation of SHP-1 targets such as Vav-1 and PLCg1 and more importantly reduces the killing of MHC-1 negative or mismatch targets. These data demonstrate that SHP-1 also plays an important role during NK cell activation and that keeping SHP-1 in an inactive conformation by PKC-θ-mediated phosphorylation is essential for NK cell-mediated anti-tumor responses. The authors use the NK cell line YTS, but also primary human NK cells sorted for KIR2DL1 expression. Overall, their claims are supported by their data. In addition to a few technical remarks, I would suggest focussing more on the role of PKC-θ-mediated SHP-1 phosphorylation during activating NK cell interactions in figures 7 and 8 by using 721-Cw7 targets for the different YTS mutants and transfectants. This would strengthen the novel point that SHP-1 plays an important role during NK cell activation.

1. In figure 7D the authors should also use the 721-Cw7 as targets for the SHP-1 ko and PKC-θ-knock-down YTS cells to demonstrate that the reduction of killing in the absence of PKC-θ is dependent of the presence of SHP-1.

As requested by the reviewer, we added a new set of experiments in which we used 721-Cw7 as targets for measuring NK cell degranulation (CD107a). As expected YTS-2DL1 cells silenced for PKCθ showed reduced degranulation compared to WT YTS-2DL1 cells following incubation with activating 721-Cw7 target cells (Figure 7E). This strengthens our results showing that PKCθ induces SHP-1 inactivation, and that upon its <milestone-start /> <milestone-end /> gene silencing, activated SHP-1 inhibits NK cell cytotoxicity. Indeed, NK cell cytotoxicity was compared between mice that were engrafted with NK SHP-1^-/-^ cells gene silenced for PKCθ versus SHP-1 S591D knock-in expressing cells. The data clearly demonstrates a restoration in NK killing potential in the SHP-1 KO cells gene silenced for PKCθ, suggesting that the reduction in killing in the absence of PKCθ is dependent on the presence of SHP-1.

2. The same applies to figure 8, where it would be more informative to see that PKC-θ-knock-down inhibits NK cell-mediated anti-tumor responses against 721-Cw7 cells and that this may be rescued by SHP-1 deletion.

As requested by the reviewers, we added a new *in-vivo* experiment in which the mice were subcutaneously engrafted with 721- Cw7 cells. As expected, mice injected with YTS-2DL1 cells silenced for PKCθ exhibit lower anti-tumor cytotoxicity, due to enhanced SHP-1 activity demonstrating higher tumor growth rate and tumor volumes compared to mice injected with WT YTS-2DL1 cells (Figure 8D and E). Furthermore, the NK cell-mediated anti-tumor responses were restored upon SHP-1 deletion.

Reviewer #3:

Ben-Shmuel and colleagues investigated the mechanisms by which the catalytic activity of SHP-I, a tyrosine phosphatase that is a critical negative NK cell regulator, is regulated during NK cell activation and inhibition. They found that activating NK cell interactions promote a rapid SHP-1 S591 phosphorylation that remains stable overtime, whereas minimal SHP-1 S591 phosphorylation was observed during induced inhibitory interactions. It is only at late time points that inhibitory signals induce high SHP-1 S591 phosphorylation. Such phosphorylation is required to inhibit SHP-1 and NK cell activity, as NK cells expressing a SHP-1 phosphor-mimetic serine to aspartic acid residue substitution (SHP-1 S591D) exhibit increased anti-tumor NK function relative to WT SHP-1-expressing cells (Ben-Shmuel et al., 2020). The authors showed by confocal microscopy an accumulation of phosphorylated SHP-1 S591 at the immunological synapse upon interaction with activating NK cell target cells. SHP-I activity is associated to conformational changes (Wang et al., 2011). The authors further used a SHP-1 Förster resonance energy transfer (FRET) bio- sensor that they have previously developed (Matalon et al., 2018), for which FRET activity is observed when the N and C terminus of the protein are closed, masking the catalytic site. FRET experiments showed that SHP-I is in a conformational inactive state at the immunological synapse as soon as the first 5 minutes of activating NK cell interactions and only after long lasting inhibitory interactions. Ben-Shmuel and coworkers further address by which mechanism SHP-I phosphorylation was achieved. Because the serine kinase PKC−θ was suggested to participate in murine NK cell activity and because PKC−θ localizes to the cytolytic NK synapse during early activation, the authors assessed its possible interplay with SHP-1 in NK cells. They showed that PKC−θ is localized at the immunological synapse early after activating NK cell contact and at late point upon inhibitory contacts. The silencing of PKC−θ limits SHP-1 S591 phosphorylation, SHP-I conformational status and enzymatic activity at the immunological synapse. Finally, they showed that PKC−θ-mediated phosphorylation of SHP-I tunes NK cell activation in vitro. in vivo, PKC−θ silencing of NK cells does not affect their anti-tumor immunity against tumor cells that engage inhibitory NK cell receptors.

The conclusions of this paper are mostly well supported by data, but one aspect need to be clarified and extended. The authors attempted to address in vivo if NK cells silenced for PKC−θ have lower anti-tumor cytotoxicity due to enhanced SHP-1 activity. However, in the final in vivo experiment they analyzed the rejection of targets cells that do not exhibit activating signals and thus for which PKC−θ-mediated SHP-I phosphorylation may not be critical. It is of most interest to evaluate the in vivo growth control of cw7 tumor cell killing by PKC−θ-silenced NK cells.

As we noted in our response to Reviewer#2, a new set of *in-vivo* experiments was added. Mice were subcutaneously engrafted with 721- Cw7 cells (which provide activating signals) and were injected with human NK cells every 72h. As expected, mice injected with YTS-2DL1 cells silenced for PKCθ exhibited lower anti-tumor activity due to enhanced SHP-1 activity, demonstrating higher tumor growth rate and tumor volumes compared to mice injected with WT YTS-2DL1 cells. (Figure 8D and E).
